# Supplementary material for: Neurodesk: An accessible, flexible, and portable data analysis environment for reproducible neuroimaging
Source: Res Sq. 2023 Mar 13:rs.3.rs-2649734. Preprint. [Version 1] doi: 10.21203/rs.3.rs-2649734/v1 (PMC10055538; doi:10.21203/rs.3.rs-2649734/v1)
Supplement: 1 [file NIHPPrs2649734v1-supplement-1.pdf]

Table S1. Differences in the execution of tissue segmentation (FIRST) and image registration (FLIRT) pipelines. Runtime refers to the CPU time spent on system and library calls within a pipeline.

	**Local**		**Neurodesk**	
**FIRST (# of calls)**	**System A**	**System B**	**System A**	**System B**
floor	553,308	553,962	553,341	553,341
floorf	48,406,500	53,942,784	51,928,356	51,928,356
log	2,820	3,138	3,024	3,024
**FLIRT (# of calls)**	**System A**	**System B**	**System A**	**System B**
floorf	41,347,920	41,334,549	41,342,544	41,342,544
**Runtime (n=8)**	**System A**	**System B**	**System A**	**System B**
Average (mins)	4.88	5.39	5.73	5.47
Standard Deviation (mins)	0.07	0.19	0.20	0.15

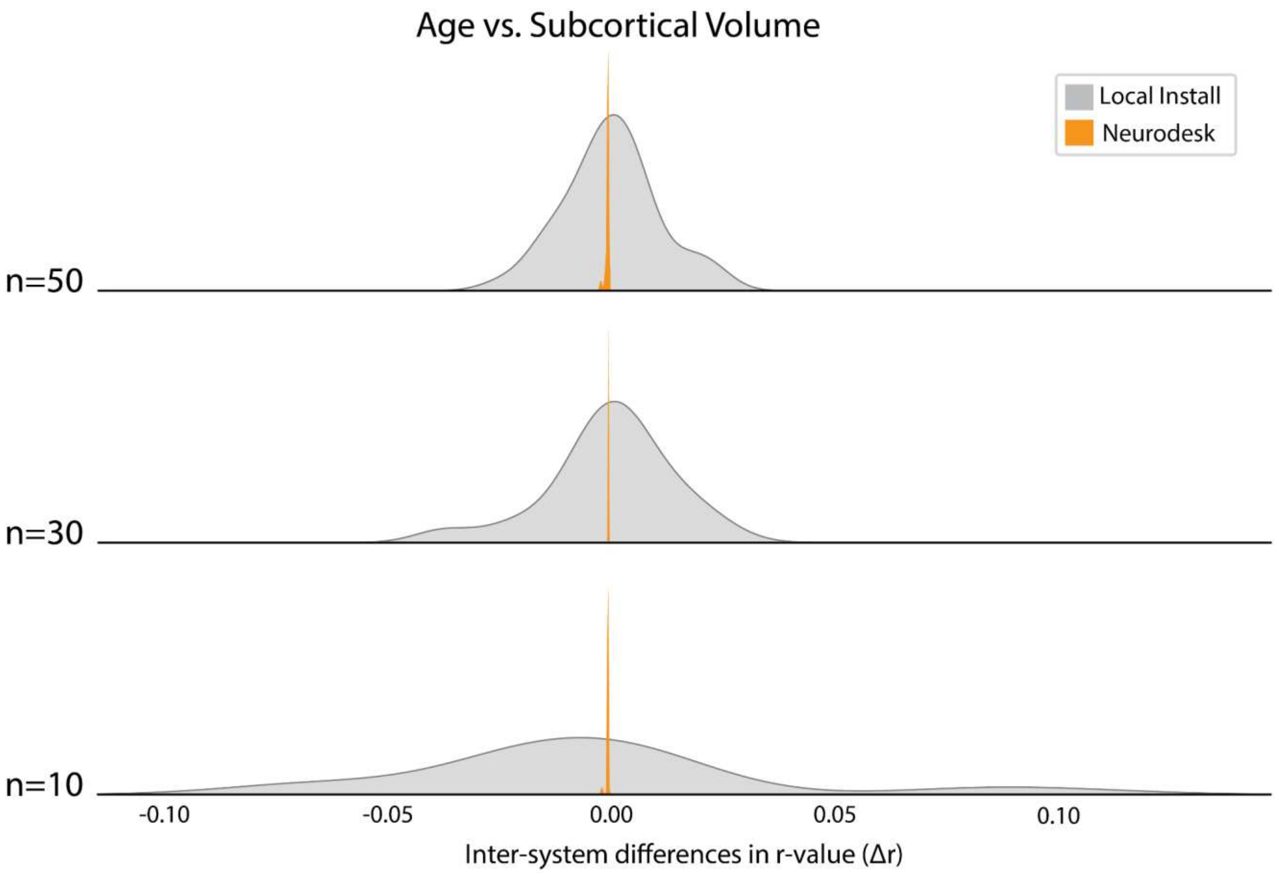

Figure S1. Permutation test results showing inter-system differences in r-values for the correlation between age and volume of subcortical structures, organized by sample size (n = 10, 30, 50).
